# Clinical Reasoning for the Examination and Physical Therapy Treatment of Temporomandibular Disorders (TMD): A Narrative Literature Review

**DOI:** 10.3390/jcm9113686

**Published:** 2020-11-17

**Authors:** César Fernández-de-las-Peñas, Harry Von Piekartz

**Affiliations:** 1 Department of Physical Therapy, Occupational Therapy, Physical Medicine and Rehabilitation, Universidad Rey Juan Carlos (URJC), Alcorcón, 28922 Madrid, Spain; 2 Cátedra Institucional en Docencia, Clínica e Investigación en Fisioterapia: Terapia Manual, Punción Seca y Ejercicio Terapéutico, Universidad Rey Juan Carlos, Alcorcón, 28922 Madrid, Spain; 3 Department of Physical Therapy and Movement and Rehabilitation Science, Faculty of Business, Management and Social Science, University of Applied Science Osnabruck, 49076 Osnabruck, Germany; h.von-piekartz@hs-osnabrueck.de; 4 Cranial Facial Therapy Academy (CRAFTA), 29097 Hamburg, Germany

**Keywords:** temporomandibular disorders, manual therapy, soft tissue, neurodynamics, needle, exercise, pain neuroscience education, grade motor imagery

## Abstract

The current narrative literature review aims to discuss clinical reasoning based on nociceptive pain mechanisms for determining the most appropriate assessment and therapeutic strategy and to identify/map the most updated scientific evidence in relation to physical therapy interventions for patients with temporomandibular disorders (TMDs). We will also propose an algorithm for clinical examination and treatment decisions and a pain model integrating current knowledge of pain neuroscience. The clinical examination of patients with TMDs should be based on nociceptive mechanisms and include the potential identification of the dominant, central, or peripheral sensitization driver. Additionally, the musculoskeletal drivers of these sensitization processes should be assessed with the aim of reproducing symptoms. Therapeutic strategies applied for managing TMDs can be grouped into tissue-based impairment treatments (bottom-up interventions) and strategies targeting the central nervous system (top-down interventions). Bottom-up strategies include joint-, soft tissue-, and nerve-targeting interventions, as well as needling therapies, whereas top-down strategies include exercises, grade motor imagery, and also pain neuroscience education. Evidence shows that the effectiveness of these interventions depends on the clinical reasoning applied, since not all strategies are equally effective for the different TMD subgroups. In fact, the presence or absence of a central sensitization driver could lead to different treatment outcomes. It seems that multimodal approaches are more effective and should be applied in patients with TMDs. The current paper also proposes a clinical decision algorithm integrating clinical diagnosis with nociceptive mechanisms for the application of the most appropriate treatment approach.

## 1. Introduction

The term temporomandibular disorder (TMD) is an umbrella generally used to describe a myriad of symptoms including muscle and joint-related pain, decreased jaw mobility, headaches, tinnitus, stiffness, fatigue, or other potential associated-symptoms [1]. 

Nassif et al. reported that almost 75% of the general population will experience TMD-associated symptoms at some point during their life [2]. TMD is associated with a substantial costs, physical and emotional burdens, and impact for society [3].

Physical therapy is probably the first therapeutic intervention requested and used by individuals with TMDs for managing their pain. A national survey conducted in the United Kingdom revealed that physical therapy is self-perceived as an effective treatment option for managing TMD-related pain [4]. Since the umbrella term TMD includes several pain conditions with different clinical manifestations but potential common underlying mechanisms, their treatment also includes different therapeutic strategies [5]. The general absence of clinical guidelines concerning physical therapy in patients with TMDs states the evident need for determining further examination and treatment decisions based on proper clinical reasoning. We conducted the current narrative literature review aiming to discuss clinical reasoning based on nociceptive mechanisms for determining the most appropriate assessment and therapeutic strategy and to identify/map the most updated scientific evidence in relation to physical therapy interventions for patients with TMDs. We will also propose an algorithm for clinical examination and treatment decisions and a pain model integrating current knowledge of pain neuroscience.

## 2. Clinical Examination Temporomandibular Pain Disorders

The examination of an individual with a TMD includes clinical outcomes such as pain intensity, pain-related disability, functional status, and kinesiophobia [6,7,8]. In the current review, we focus on the assessment of pain mechanisms. In such a scenario, classifying or subgrouping patients according to phenotypes (i.e., observable characteristics, personal traits, or clinical presentation without mechanistic implications) and also endotypes (i.e., determining the particular diagnosis and the underlying pathophysiological mechanisms) would permit us to better characterize and select therapeutic approaches. The identification of features of peripheral and central sensitization could help us to characterize chronic pain patients to more homogenous psychopathological profiles and also to determine potential treatment strategies [9].

### 2.1. Identification of Central Sensitization

Clinical indicators of central sensitization include widespread pain hyperalgesia and allodynia, the absence of conditioned pain modulation, sleep disturbances, and comorbid psychological disorders. The presence of pain hyperalgesia to different stimuli has been traditionally assessed, mostly in research practice, using quantitative sensory testing. Quantitative sensory testing assesses homosynaptic and heterosynaptic mechanisms of sensitization and includes the application of different stimuli such as vibration, thermal, electrical, and mechanical in symptomatic and distant pain-free areas [10]. It is important to note that quantitative sensory testing is not a diagnostic test for a particular condition; it is a tool for helping in the mechanism-based diagnosis of pain mechanisms [11]. There is evidence supporting the presence of an altered nociceptive processing in patients with TMDs expressed by widespread lower pain thresholds to mechanical but not thermal stimuli [12]. In fact, widespread pressure hypersensitivity seems to be a common feature of musculoskeletal pain conditions of the head-neck region, such as TMD [12], or tension-type headaches [13].

Since quantitative sensory testing is not available for regular clinical setting, patient-reported measures assessing central sensitivity related-symptoms are also proposed. The Central Sensitization Inventory (CSI) is a self-reported questionnaire developed for identifying central sensitization-related symptoms [14]. It has been established that scores greater than 40 points are suggestive of central sensitization [14]. Neblett et al. identified that patients with TMD (23% of the subjects attending an outpatient chronic pain clinic) exhibited scores of the CSI suggestive of central sensitization [15].

Although the CSI is used as a potential clinical tool for identifying the presence of central sensitization, relying on the exclusive use of patient-reported measures such as the CSI for identifying central sensitization appears to be challenging. A combination of patient-reported measures with psychophysiological data such as quantitative sensory testing is recommended. 

In addition, it seems appropriate to position the identification of central sensitization as a prognostic rather than a diagnostic tool.

This proposal is supported by recent evidence showing that the presence of central sensitization patient-reported measures is a good predictive tool in musculoskeletal pain conditions, since more severe central sensitization related-symptoms predict higher pain-related disability in individuals attending primary care [16], but also impaired quantitative sensory testing is able to predict worse pain intensity and related disability [17].

It is important to consider that the presence of central sensitization should not prevent clinicians from searching for peripheral tissue impairments in the context of a conditions model that takes into account both sensitization processes. The next sections of this review describe the clinical examination of peripheral structures in individuals with TMDs focusing on nociceptive pain mechanisms.

### 2.2. Manual Palpation

Palpation is probably one of the most relevant clinical examination tests because of its high inter-reliability and the structural differentiation within the tissue painful tissue [18]. Similarly, if there is predominantly muscle pain, the clinician can explore which muscle is most painful (involved) and which part of the muscle. This reasoning is equal for joint and nerve structures.

#### 2.2.1. Joint Palpation

From clinical studies, it is stated that the posterior pole of the temporomandibular joint (TMJ) is the most sensitive area in patients with arthrogenic TMD [19]. In fact, pain with joint palpation in elderly people is able to discriminate (non) TMD [20]. Palpation during a (sub) acute arthrogenic TMD may be highly sensitive and, in these cases, the pain threshold is an indication to stop. [21] An optimal pressure for differentiating the joint structure with a moderate to good specificity (89.7%) and sensitivity (70%) was found to be 1.4 kg/cm^2^ of pressure [22]. Duration in palpation is, according to the authors, not clear, but from clinical experience less than 2–3 s is enough to obtain appropriate information. A recent study found that the Rocabado pain map, which is a structural palpation differentiation test of eight structures, is a reproducible and reliable palpation set, mostly in the synovial and ligaments zones (ICC values from 0.64 to 0.84), but does not differentiate between hyper and hypomobility [23].

#### 2.2.2. Muscle Palpation

In healthy subjects, normal pain thresholds on the masticatory muscles have been estimated to be around 1.5–2.0 kg/cm^2^ (extra-oral) and 1 kg/cm^2^ (intra-oral) [24]. Muscle palpation can be measured with a pressure algometer or with the fingertips; both procedures have shown an excellent inter-rater reliability [25]. The masticatory muscles exhibit lower pain thresholds—e.g., <1 kg/cm^2^ intra-oral—which, in the clinical setting, make it often easy to distinguish patients with myofascial TMD [26]. One sign is tenderness with manual palpation, whereas a different sign is the presence of muscle pain referral elicited by trigger points [27]. The diagnostic criteria for temporomandibular disorders (DC/TMD) determine that the palpation of the masticatory musculature should be considered positive when a patient recognizes the palpation-induced pain as a familiar pain—i.e., pain recognition [28]—since the presence of tenderness in a specific tissue could reflect hyperalgesia and/or allodynia. The DC/TMD recommends the application of 1 kg of pressure for 2 s for the diagnosis of muscle pain (myalgia subtype) [28]. If the clinician wants to elicit referred pain during the palpation, the pressure can be maintained for up to 5 s [28]. The presence of trigger points may influence orofacial motor control, as expressed in loss of movement, muscle weakness, muscle inhibition, or accelerated fatigability [29]. A proper distinction between tenderness and trigger point seems to be relevant.

#### 2.2.3. Nerve (Trunk) Palpation

As already established within the lower and upper extremities, the classification of neural tissue disorders into subgroups permits identifying potential patients suitable for neural mobilization treatment. This subclassification system based on the pathobiological mechanism and dysfunction of nerve trunk tissue requires a comprehensive examination process. In the head-neck and face pain region, a possible order of diagnosis suggested for this subclassification is Neuropathic Pain with Sensory Hypersensitivity (NPSH), Compression Neuropathy (CN), or Peripheral Nerve Sensitization (PNS); in the last one there would be no neurogenic involvement—i.e., Musculoskeletal Pain (MP) [30].

Self-reported screening tools for identifying a neuropathic/neurogenic pain component—e.g., the Leeds Assessment of Neuropathic Symptoms and Signs (LANSS) Questionnaire [31] or the Pain DETECT [32]—are available. Both questionnaires are highly valid and have been translated into different languages. For instance, the PainDETECT has been identified as a bedside measure for patient stratification to better predict treatment outcomes in osteoarthritis patients undergoing exercise [33]. A positive LANSS (>12 points) or Pain DETECT (>19 points) suggests an NPSH dominance, possibly caused by the sprouting of nerve branches eliciting spontaneous shooting pain and sensory changes such as hypo-hyperesthesia or dysesthesia. In these cases, nerve trunk palpation would be less appropriate due to the greater potential for nerve lesion than in those cases with more CN and PNS dominance where nerve trunk-related pain is more involved.

The provocation of symptomatic complaints during nerve palpation suggests a potential role of nerve tissue but does not necessarily identify the site of neural tissue injury, because the entire neural tissue tract can become mechanically sensitive [34,35]. Symptoms can be evoked by the palpation of the mental branch in the mental foramen on the chin [36] or the auriculotemporal nerve [37]. Clinically, the mandibular branches, which include the lingual, inferior alveolar, mental, and auriculotemporal nerve, are potentially palpable, particularly in their neurodynamic positions; they, together with the other cranial nervous tissue, provide an impression of pain from neurogenic TMD. Palpating the nerve with the tip of the finger or thumb is the easiest technique. Once a nerve is identified, place the tip of the finger lateral of the nerve, move in transverse, and let it move it under your finger at a high speed. This technique is called “twanging” and is, according to the clinical experience of the authors, the most appropriate technique for the palpation of cranial nerves [30].

### 2.3. Musculoskeletal Tests

In general, musculoskeletal tests are characterized by a consistent lack of diagnostic standards. They are furthermore influenced by several therapist and patient-related aspects and have a large variation in neuromusculoskeletal signs and symptoms. Therefore, clinical diagnosis should not be based on just one test only. The clustering of relevant musculoskeletal tests related to the subjective examination and multi-test scores (combination of several pain-related variables) increases the diagnostic value. An example of positive multi-test scores is when, during the test procedure, the signs brought up one by one or when several parts of a clinical test confirm a clinical pattern [38]. For example, if TMJ noises are provoked during active movements AND an additional test such as a static test is positive, then one can conclude that the applied multi-test score is positive. Table 1 shows the results of the inter-examiner reliability of multi-test scores for most cardinal symptoms of TMD: pain, noise, and movement restriction [38]. It may be possible to confirm the hypothesized clinical pattern or diagnosis using clustered multi-test scores, and this can then be the clinical starting point for management. For example, when two clinical tests show pain and the restriction of movement and the tests for noise are negative, the hypothesis of a myogenic TMD could be made.

After a subjective examination including current and previous history, orofacial behaviors, and predictive/contributing factors, the clinician decides to use a cluster of relevant clinical tests which match the adequate reliability of the multi-test scores (k-values > 0.4) [38,39]. The clustering of musculoskeletal tests will be even more efficient during reassessment after the treatment, when the implemented tests also measure clinically relevant changes—i.e., minimal detectable change (MDC) or minimal clinical important difference (MCID) [40]. Unfortunately, it has to be mentioned that not all orofacial clinical tests have defined these psychometric properties.

The combination of subjective examination and a clinical test, which has a moderate to high reliability (k-values > 0.4), provides clinicians with the possibility to determine a first hypothesis for subclassifying into arthrogenic, myogenic, or neurogenic TMD based on an impression of the cardinal signs and symptoms of TMD-related pain. Steenks et al. analyzed a sample of 160 patients with TMD and their classification according to different combinations of tests and were able to differentiate between myogenic (*n* = 69) and arthrogenic (*n* = 91) TMD [38]. Unfortunately, in actual dentistry models neurogenic TMD is not accepted as its own clinical subclassification in the RD/TMD.

A recent Delphi survey with a panel of 25 international experts identified eight physical examination tests for the evaluation of TMD: physiological temporomandibular joint (TMJ) movements, the trigger point palpation of the masticatory muscles, trigger point palpation away from the masticatory system, accessory movements, joint palpation, noise detection during movement, the manual screening of the cervical spine, and the neck flexor muscle endurance test [41]. Most clinical experts (*n* = 21, 84%) combine the test to subclassify into dominantly arthrogenic, myogenic, or neurogenic TMD [41].

#### 2.3.1. Physiological Movements

For instance, a combination of physiological active movements and passive movements (mouth opening, lateral deviation, retrusion, and protrusion) related to the multi-test scores may give a good sense of TMD, and its potential subclassification as the combination of changes in a range of movement and pain may give the clinician an impression of which tissue is involved and needs more detailed examination. For example, it is known that there is a ratio 4:1 between mouth opening and lateral deviation—e.g., 50 mm of mouth opening to 12 mm of lateral deviation. A patient showing lateral deviation toward one side consistently restricted more than 5 mm combined with a mouth opening restriction with a deviation towards one side is indicative of an intra-articular TMJ dysfunction.

#### 2.3.2. Accessory Movements

**Figure 1:**
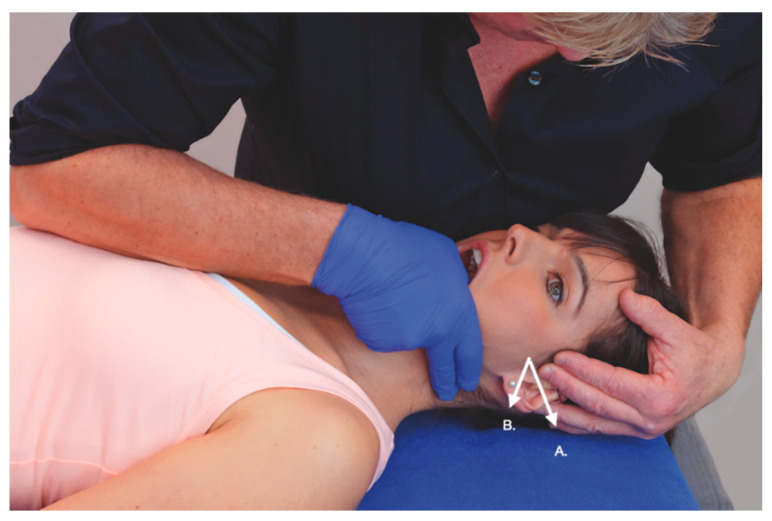

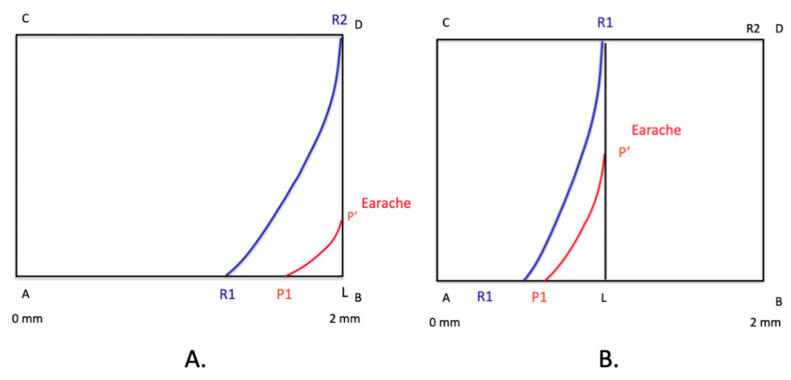
Accessory movement in the transverse sliding of the TMJ (temporomandibular joint) towards lateral (**A**) and with an angulation towards 30° posterior (**B**). Movement diagram A shows exponential curve (R1-R2) with no limitation when the average movement excursion is 2 mm. The reproduction of ear pain is minimal (P1-P”) (2/10 on a numerical pain rate scale, NPRS). Movement diagram B shows the angulated transverse to lateral, where R1-R2 is more than 50% restricted and the ear pain is stronger (6/10 NPRS), suggestive of lateral pterygoid muscle involvement. Reprinted with permission from Physikalisch Untersuchung von Dysfunktionen der kraniomandibulären Region. In: von Piekartz HJM (ed). 2nd edition. Kiefer-Gesichts-und Zervikalregion. Auflage: Thieme, 2015. Pg 212.

Accessory movements consist of passive maneuvers, including the gliding/sliding of the articular surfaces, with a particular direction aimed to assess parameters such as resistance, spasm, and sensory responses [42]. Accessory movements such as longitudinal caudal, cranial, medial, and lateral transverse, antero-posterior, and posterior-anterior with angulation and combinations are particularly considered for peri- and intra-articular structures; therefore, they can help in hypothesizing differentiation between structures. For example, the transversal to lateral sliding has an increased resistance and reproduces ear pain in a patient with TMD. By angulating the transverse sliding to lateral with 30° towards dorsally, ear pain increases and resistance starts earlier; this would suggest the involvement of the lateral pterygoid muscle (Figure 1). Similarly, an increase in ear pain during upper cervical flexion and lateral flexion away for the symptoms side may be partly caused by the peripheral nerve sensitivity of the mandibular nerve [43].

#### 2.3.3. Neurodynamic Testing

**Figure 2:**
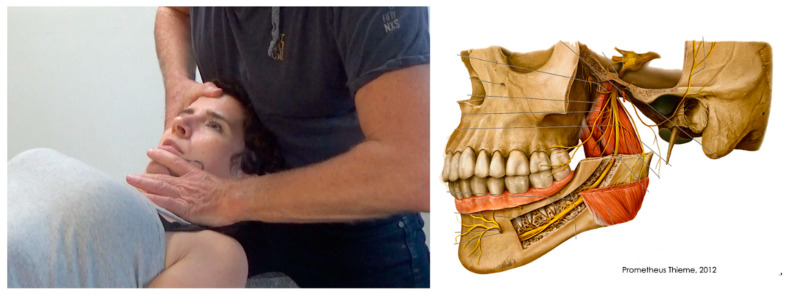
Performance of the neurodynamic test of the mandibular nerve: upper cervical flexion, upper lateral flexion toward the contra-lateral side, mandible lateral deviation in 2.5 cm mouth opening to the contra-lateral side. Right figure reprinted with permission from Hals und Neuroanatomie. In: Prometeus K (ed). 4th edition. Auflage: Thieme; 2015. Pg. 123.

In addition to nerve palpation, the clinical testing of nerve mechano-sensitivity in a patient with a PNS dominance needs to include a neurodynamic test—i.e., a specific sequence of movements mostly related to the nerve trunk anatomy. It is evident that a positive test may relate to non-neural and neural tissues and does not identify the site of the potential neural tissue injury [44]. For instance, within the temporomandibular region, the oval foramen of the skull floor, the head of the mandible, or the lateral pterygoid are anatomically in contact with the mandibular nerve branch [45]. These structures have to be examined regarding their influence on cranial neural tissue during normal head and mandibular movements. The decrease in potential compressive pressure on the nerve from these structures may improve conductive function and decrease neural mechano-sensitivity [43,46]. Minor studies on the movement of cranial nerve tissue exist, but they are mostly described in the (suboccipital) neurosurgery and maxillofacial literature. Doursounian et al. [47], using Magnetic Resonance Imaging scans, found that the spinomedullar angle changes from 6° to 32° when the upper cervical spine moved from neutral to flexion. This confirms the results of Breig [48], who also reported that the upper lateral flexion of the head challenges cranial nervous system movement in the trigeminal nerve. Mouth opening and lateral deviation towards the contra-lateral side may put more load on the lingual [49] or inferior alveolar [50] branches. Cervical flexion and the longitudinal movement of the mandibula causes the movement of the auriculotemporal nerve [51]. Therefore, based on current evidence, for example the examination sequence for the mandibular nerve could be a combination of cranio-cervical flexion and lateral flexion to the contra-lateral side in about 25 mm mouth opening (Figure 2). During this movement, the mandibular nerve may cause a maximal excursion of movement towards the contra-lateral side (lateral deviation) without maximal stress on the intra articular TMJ tissue [43].

A case report showed that the assessment and manual treatment of the auriculotemporal nerve and its interface lead to pain relief and improvements in orofacial function [52].

## 3. Integrating Bottom-Up and Top-Down Interventions

The therapeutic management of chronic pain syndromes such as TMDs should focus on nociceptive pain mechanisms instead of biomechanical models [53]. Although there is a debate about the role of peripheral or central mechanisms, evidence supports the notion that central sensitization seems to be initiated by long-lasting and prolonged peripheral nociception (peripheral sensitization); however, once central sensitization has been established, only minimal peripheral nociception is required [54]. Therefore, while it is logical to target the central nervous system to reduce central sensitization, it has to be stressed that those treatments aiming to reduce peripheral nociception can potentially attenuate central sensitization in an undetermined proportion of patients. It is clinically seen that both peripheral and central mechanisms create a vicious circle in patients with TMDs.

Pfau et al. identified two subgroups of patients with TMD based on sensitization mechanisms—a sensitive group exhibiting more central sensitization features and a non-sensitive group showing more peripheral sensitization features [55]. The identification of the predominant sensitization mechanism, more peripheral or more central can be highly relevant since central sensitization is a predictor of poor clinical outcomes in some, but not all, musculoskeletal pain conditions [56]. The role of central sensitization as prognosis factor for a specific treatment has been researched in other conditions, but not in TMD. For instance, central sensitization predicts poor response for exercise and pain education in patients with knee osteoarthritis [57], or following injection treatment for chronic lateral epicondilalgia [58]. Additionally, the presence of higher or lesser sensitization could also determine the dosage—e.g., intensity, amplitude, or frequency of the treatment sessions—of the intervention. Therefore, identification of the dominant sensitization mechanisms in patients with TMD is required for a proper clinical reasoning in physical therapy [59].

In such a scenario, the challenge for clinicians is how to select proper treatment for each individual with TMD who is likely to present a particular clinical presentation. An important topic for determining the proper therapeutic strategy is to determine if the clinical presentation of a patient with TMD is more peripheral or more central dominant [9].

From a clinical perspective, in individuals primarily mediated by peripheral inputs (bottom-up sensitizers), therapeutic strategies targeting tissues potentially related to the peripheral nociception—e.g., muscle, articular or nerve tissues, combined with segmental exercises targeting the specific musculoskeletal disorders associated with TMD—should be encouraged. In individuals primarily mediated by potential central processes (top-down sensitizers), therapeutic strategies should extend beyond tissue-based impairments to incorporate interventions aimed to normalizing the excitability of the central nervous system—e.g., psychological or cognitive approaches, pain education, and more general exercise programs [60]. In fact, this hypothesis agrees with the current understanding of those underlying pain mechanisms explaining the effects of manual therapies [61] and needling [62] interventions. In fact, both models integrate bottom-up and top-down interventions considering the context, the patient’s expectances and previous experiences, and the placebo effect [61,62].

## 4. Scientific Evidence of Bottom-Up Interventions

Bottom-up interventions are those acting on the central nervous system by applying a peripheral stimulus on a specific tissue—i.e., muscle, articular, or nerve trunk. The most common bottom-up therapeutic strategies used for the management of individuals with TMD are manual therapy and dry needling. Interventions targeting nerve trunk related-pain—i.e., neurodynamics—are less used in this condition, but will be also discussed.

### 4.1. Manual Therapies

The term manual therapy includes a large number of hands-on interventions ranging from joint-biased interventions (joint mobilizations and/or manipulations) or soft tissue-biased interventions (muscle stretching or trigger point pressure release) to therapeutic exercises [63]. In addition, nerve-biased interventions, also called neurodynamic treatments, should be also included in this term.

**Figure 3:**
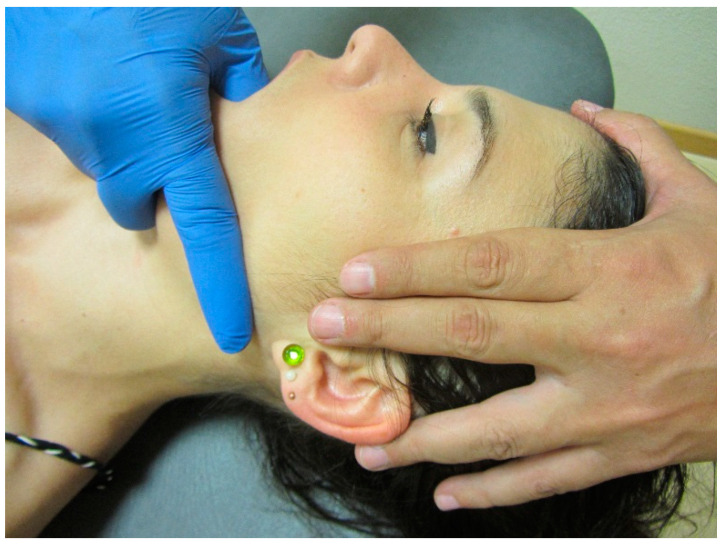
Mandibular distraction mobilisation technique. Copyright Handspring Publishing Limited 2018. Reproduced with permission. Illustrations first published in Temporomandibular Disorders edited by César Fernández-de-las-Peñas and Juan Mesa-Jiménez, Handspring Publishing Limited, 2018.

**Figure 4:**
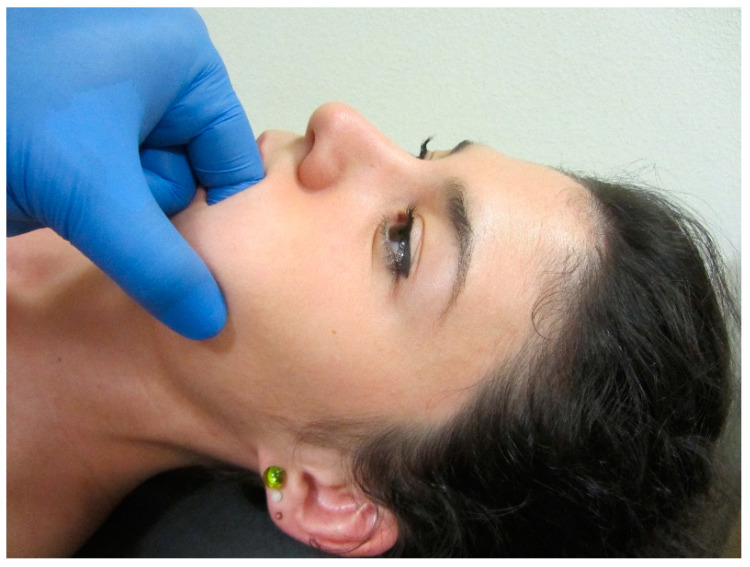
Intra-oral pincer compression of masseter muscle. Copyright Handspring Publishing Limited 2018. Reproduced with permission. Illustrations first published in Temporomandibular Disorders edited by César Fernández-de-las-Peñas and Juan Mesa-Jiménez, Handspring Publishing Limited, 2018.

**Figure 5:**
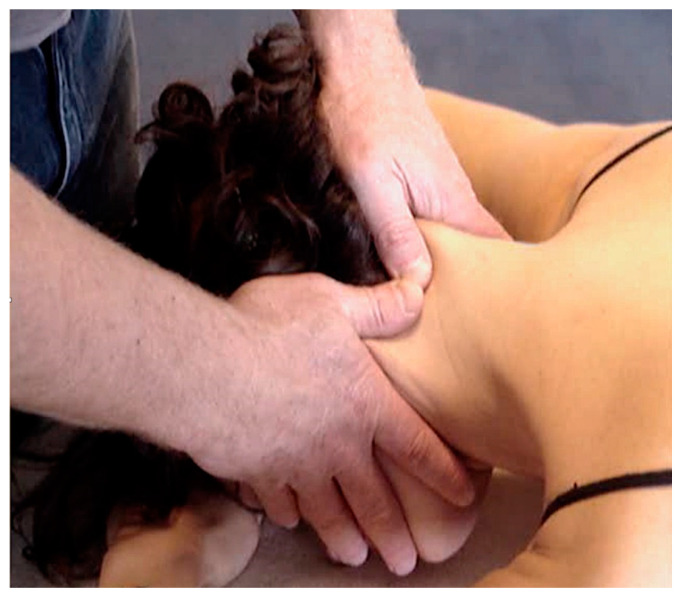
Unilateral posterior-anterior mobilization of the upper cervical spine.

Several manual therapies are clinically applied for the management of TMDs. The rational for the application of joint-, soft tissue- or nerve- biased interventions is the presence of musculoskeletal disorders in individuals with TMDs. In fact, patients with TMD are treated with manual therapies directly targeting the temporomandibular joint (Figure 3), masticatory musculature (Figure 4), or cranial neural tissues (Figure 2) depending on the main structure responsible of nociception (previous section). Further, due to the relationship between the upper cervical spine and the temporomandibular joint, manual therapies targeting the upper cervical spine are also proposed (Figure 5).

Several systematic reviews have investigated the effects of manual therapies for the treatment of TMD. Calixtre et al. found low to moderate evidence supporting that myofascial release and massage therapy applied to the masticatory muscles and spinal manipulative therapy applied to the upper cervical spine are more effective than control for the management of TMD pain [64]. Armijo et al. concluded that manual therapy alone or combined with exercises showed promising benefits for treatment of TMD, although the effect sizes were low to moderate and related to the particular type of TMD [65]. For instance, manual therapy was effective for reducing pain in the short term compared with Botulinum toxin or waiting list in myofascial TMD, but not in arthrogenic TMD [18].

Martin et al. found large effects for decreasing pain during active mouth opening in favor of manual techniques when compared to other conservative treatments for TMD [66]. Similarly, the meta-analysis by Paco et al. found that physical therapy was more effective for reducing pain intensity but equally effective for improving the active range of movement than the control group [67]. 

The discrepancies in the results from these reviews can be related to the hypothesis that different TMD groups—e.g., myofascial or arthrogenic—probably will need different manual therapy approaches; therefore, determining which particular manual therapy can be applied to which TMD subgroup is highly relevant.

In agreement with this reasoning, two recent systematic reviews have analyzed the effects of manual therapies according to TMD subgroups. La Touche et al. found preliminary evidence suggesting that manual therapy and therapeutic exercise may be beneficial for the management of patients with disc displacement without reduction [68]. Similarly, de Melo reported that manual therapy was as effective as botulinum toxin or counseling for decreasing pain in patients with myofascial TMD [69]. A proper clinical reasoning determining which manual therapy intervention should be better applied to a particular patient is clearly needed. Moreover, some patients with TMD could also benefit from manual therapy targeting the cervical spine. A recent meta-analysis reported that manual therapy targeting the cervical spine is effective for decreasing pain intensity and increasing the range of motion in patients with TMD (low to moderate evidence) [70].

Although current evidence clearly supports that the application of manual therapies can be effective for TMD, it is not possible to draw any firm conclusion about the most effective techniques to improve pain and the range of motion. This is related to the fact that most published studies have investigated the isolated effects of particular interventions. It is important to consider that patients with TMD need multimodal approaches and not isolated strategies. There is preliminary evidence supporting the combination of manual therapy, education, and exercises for patients with myofascial TMD [71,72], arthrogenic TMD [73], anterior disc displacement without reduction [74], or cervico-craniofacial pain [75].

### 4.2. Needling Interventions

Needling therapies consist of wet (medical doctors) and dry (physical therapists) interventions and are commonly used for the management of TMD-related symptoms. Wet needling (injections) includes the application of pharmacological substances—e.g., local anesthetics or botulinum toxin—whereas dry needling uses filament solid needles without the application of any substance. Depending on the clinical reasoning used during the application of needles, we can differentiate between acupuncture or trigger point dry needling. The American Physical Therapy Association (APTA) defines dry needling as “skilled intervention using a thin filiform needle to penetrate the skin that stimulates trigger points, muscle, or connective tissue for the management of musculoskeletal pain disorders” [76]. Clinical rational for the application of dry needling would be related to the fact that trigger points in the head and neck muscles are able to reproduce the symptoms in individuals with TMD [77].

**Figure 6:**
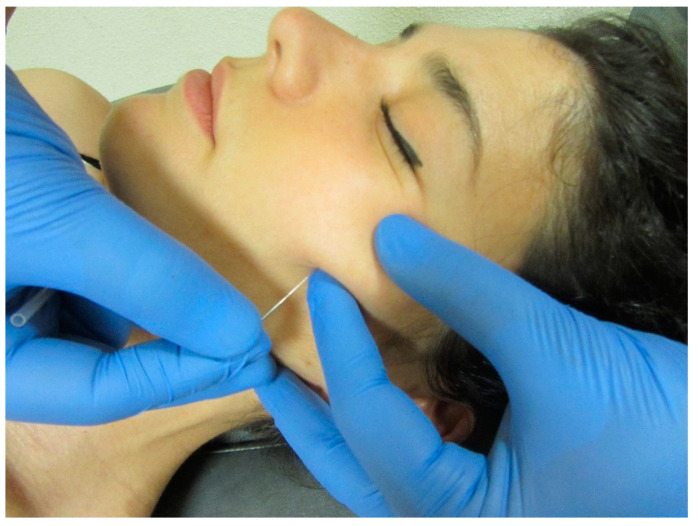
Dry needling of the zygomatic muscle (pincer palpation). Copyright Handspring Publishing Limited 2018. Reproduced with permission. Illustrations first published in Temporomandibular Disorders edited by César Fernández-de-las-Peñas and Juan Mesa-Jiménez, Handspring Publishing Limited, 2018.

Single studies have reported that the trigger point dry needling of masticatory muscles (Figure 6), is effective for reducing pain symptoms in people with myofascial TMD [78] or sleep bruxism [79]. In fact, dry needling has shown better clinical outcomes than pharmacological drug treatment [80].

A recent meta-analysis has concluded that dry needling is effective for reducing pain intensity compared with other interventions; however, the data were based on low-quality trials (high risk of bias) [81]. Similarly, acupuncture appears to be effective for relieving pain symptoms in individuals with myofascial TMD, although the level of evidence is also low [82].

It is important to discuss that the effect of needling interventions mainly comes from the mechanical effect of the needle and it is not mostly associated with the substance associated. This is supported by several studies demonstrating similar clinical outcomes after applying trigger point dry needling, injections of lidocaine or Botulinum toxin A in patients with TMD [83,84]. This hypothesis is supported by a recent network meta-analysis concluding that the effectiveness of needling therapies did not depend on the needling type (dry or wet) or needling substance [85]. It seems that needling therapies could be effective for some individuals with TMD; however, the number and frequency of sessions, dosage, and which muscles should be needled remain unknown.

## 5. Scientific Evidence of Top-Down Interventions

Top-down interventions claim to act directly on the central nervous system without the application of a specific peripheral stimulus. The most common top-down therapeutic strategies used for the treatment of patients with TMD include therapeutic exercise, pain neuroscience education, and grade motor imagery.

Although exercise-induced hypoalgesia acts mainly by activating the descending inhibitory pain pathways [86], it is also claimed that manual therapies elicit analgesic effects that are at least partially mediated by the same mechanisms, descending modulation [87]. A recent meta-analysis found that physical therapy is able to modulate central sensitization-related variables by decreasing the temporal summation and increasing the conditioned pain modulation in patients with musculoskeletal pain, confirming that not only top-down but also bottom-up interventions are able to activate descending pain modulation [88].

### 5.1. Therapeutic Exercise

Therapeutic exercise is of the main top-down intervention used for the treatment of chronic pain [89], and its inclusion in the management of patients with TMD is currently promoted. Clinicians should differentiate between segmental local exercises—i.e., those targeting specific musculoskeletal disorders of the cranio-cervical and orofacial regions—and aerobic exercises—i.e., those targeting global health wellness. This differentiation is highly relevant, since the underlying mechanisms and the objectives of each type of exercise are different. For instance, localized exercises aim to address specific impairments and promote the proper function of the cranio-cervico-mandibular system. Experts participating in a recent Delphi study concluded that patients with TMD should always be instructed in individualized jaw exercise programs and also receive verbal and written instructions about the exercise program modality [90]. Localized orofacial exercises should be targeted to improve muscle coordination, relax hypertonic muscles, increase the range of motion, and increase muscle proprioception and muscle endurance [91]. These exercise programs can be supervised or home-based, where the participation of the patient plays a relevant role. The systematic review by Dickerson et al. found that mobility and mixed exercises of the cranio-cervical area are the most common exercise regimes used for the management of TMD-related pain, and both provide positive effects on pain and jaw range of motion; however, no consensus exists on which approach is the most effective [92]. Additionally, no info about appropriate dosage, frequency, or adherence to exercise is available.

In addition to localized exercises, general aerobic exercises should be also promoted for promoting well-wearing and better health status. Nevertheless, it is important to note that patients exhibiting central sensitization, such as TMD, can exhibit an abnormal pain response to exercise since aerobic exercise usually exerts exercise-related hypoalgesia by activating the descending inhibitory pain mechanisms. In individuals with TMD with central sensitization, exercise could induce hyperalgesia [93]. In fact, it has been found that the application of aerobic (isotonic) exercise alone was not effective in reducing TMD-related pain [94].

Considering the minimal side effects of general exercise, individuals with TMDs should be encouraged to practice physical exercise with proper intensity, frequency, and duration to achieve the most beneficial clinical outcomes. In fact, there is a lack of evidence as to what type of exercise, intensity, and dosage should be administered to treat TMD. Another relevant topic is adherence and patient satisfaction. These aspects are intrinsically linked to the development of pain during exercise. For instance, inappropriate exercise may induce hyperalgesia if not properly controlled; therefore, aggressive or excessive exercise programs may be detrimental for patients with TMD if the intervention triggers peripheral nociception and promotes pain. Therefore, inappropriate exercise programs could worsen the clinical scenario of a patients with TMD if not properly controlled.

### 5.2. Pain Neuroscience Education

The difficulty in the proper long-term management of individuals with chronic pain usually lies in the complex task of changing the attitudes, lifestyles, and also social and physical environments of the individual. This assumption is based on the hypothesis that pain is clearly influenced by inappropriate cognition, emotions, and behaviors, including catastrophizing, hyper-vigilance, avoidance behavior, and somatization. It has been seen that patient education using biomedical models does not only fall short in helping to relieve pain and pain-related disability but may in fact induce fear, which increases the pain experience [95].

Psychological disturbances and mood disorders should be treated by the appropriate professional—i.e., a psychologist; however, pain neuroscience education can be applied by other health care professionals—e.g., physiotherapists. Therapeutic pain neuroscience education can be defined as “educational sessions, directed by health care professionals, providing patients’ basic and lay knowledge about potential underlying mechanisms of their condition and proper skills to manage their lives” [96]. There is preliminary evidence suggesting that neurophysiology education aiming at conceptualizing pain, reducing fear, and convincing the patient of their own control over pain and movement can be effective in people with chronic pain, and it is recommended that this is included in the initial treatment phase of those patients exhibiting inappropriate beliefs about their pain symptoms and complaints [97]. An early identification of these factors would be highly important, since a poor understanding of the pain experience by the patient may lead to the acquisition of maladaptive attitudes, cognitions, and behaviors and a consequent poor compliance to any active exercise program. Kalamir et al. found that the inclusion of short talks covering the anatomy, physiology, and biomechanics of the jaw plus the instruction and supervision of self-care exercises increased the effects of myofascial therapies in patients with TMD in the short and long term [98]. Gokhale et al. show that applying pain neuroscience education in one-to-one or in groups was equally effective when combined with physical therapy in individuals with TMDs [99]. A recent mixed methods study combining manual therapy, pain neuroscience education, and exercises applied by a special app was effective in reducing face pain and increasing function and quality of life [100]. Interestingly, it seems that the positive effects of pain neuroscience education for improving kinesiophobia and illness beliefs in patients with chronic pain are not related to the presence or absence of central sensitization [101].

Nevertheless, recent evidence suggests that pain neuroscience education evokes small effects on pain and pain-related disability and moderate effects on pain catastrophizing and kinesiophobia [102]. Similarly, the combination of pain neuroscience education with other interventions does not have more positive effects than the isolated application of the other intervention in chronic musculoskeletal spinal pain [103,104]. It is probably the case that pain neuroscience education should be effective in a subgroup of patients, but not all.

### 5.3. Graded Motor Imagery and (Face) Emotion Expression Exercises

It is also important to note that the TMD may be associated with body (face) disruption, which can be expressed as alexithymia (a lack of emotional expression and emotional recognition), changes in two-point discrimination, and (facial) motor disturbances [105,106,107]. This situation may lead to facial dysmorphic disorders (dysfunctional body image, in this case of the subject’s face) [108] or prosopagnosia (a severe deficit in recognizing familiar people from their face) [109]. Because of these complex changes in neural brain connectomes (comprehensive map of neural connections in the brain), the pain matrix maybe disrupted, contributing hence to chronic pain [110]. Body image changes in patients with TMDs may be observed quickly, which is hypothesized by the large somatosensory projection of the face on the brain. Therefore, the assessment and treatment of the brain—e.g., with graded motor imagery (GMI) and (basic) emotion expression training—could strongly affect pain experience and increase functional activities [111]. If the clinician decides to apply brain training in a TMD patient, the next progression is suggested.

**Figure 7:**
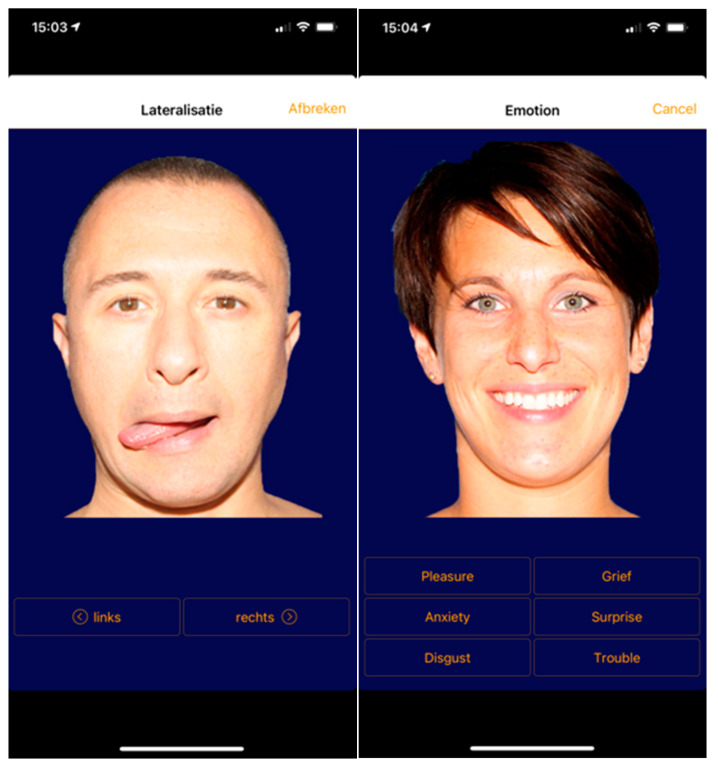
Example of the laterality test (**left image**) and basic emotion test training (**right image**) on a mobile telephone, which may be converted in intervention parameters easily.

First, left/right judgements (laterality retraining) are checked, where the patient has to identify in pictures or a special app the model’s eye, tongue, eyebrow, or jaw as moving on or towards either the left or right side of their face as soon as possible. Accuracy and time can be measured. If the accuracy (%) and average time (sec) of the left/right recognition is equal, GMI (mental representation without physic movements) can be implemented to the previous left/right exercises. If symptoms are not increased with this training, the patient is invited to express the face movements without pain with mirror therapy, whereby the healthy part of the patient’s face is used to display a second part for reinforcing the process of motor imagery [112]. In fact, disrupted motor processing manifested as insufficient left-right discrimination may underpin and contribute to facial pain and emotion recognition difficulty [106]. Therefore, the next step would be (basic) emotion recognition and facial expression training, where different software can be used at www.myfacetraining.com (Figure 7).

## 6. Algorithm for the Clinical Examination and Treatment of TMD

**Figure 8:**
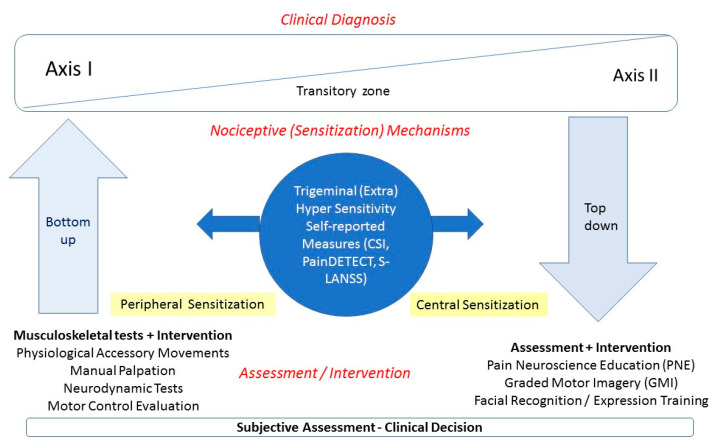
Algorithm for the clinical examination and treatment of patients with TMD based on nociceptive pain mechanisms.

Figure 8 shows a proposal algorithm based on the clinical reasoning discussed in the current review. After subjective assessment during the clinical history of a patient with TMDs, the clinician has to reflect on which data support the predominant nociceptive mechanism, central or peripheral, and must decide which region will be particularly examined.

Clinical examination with musculoskeletal tests can generate information on which sources may be dominantly involved in inducing peripheral sensitization (trigeminal sensitivity) and contributing to Axis I (arthrogenic, myogenic, combined with/without neurogenic component). For instance, in a patient with myogenic TMD, the palpation of the masticatory muscles can reveal the presence of muscle pain, just localized or referral, but not joint pain. On the opposite side, in a patient with arthrogenic TMD disorder joint palpation should be able to reproduce the symptoms from the patient, whereas muscle does not. The identification of the primary peripheral driver during clinical examination would lead to the application of one or other bottom-up approach—e.g., dry needling or localized exercise.

If the symptoms suggest that central sensitization is the dominant driver in a particular patient, psychological aspects (Axis II) should be highly taken into account. In fact, clinicians should be aware that symptom complaints can manifest dynamically over time and often have overlaps (transitory zone). In TMD patients with a central sensitization predominant mechanism, top-down approaches should be applied.

## Figures and Tables

**Table 1:** Inter-examiner reliability of musculoskeletal tests related to the three main symptoms of TMD (adapted from Steenks et al., 2007).

Musculoskeletal Tests	Agreement%	K	Presence of Signs and Symptoms,%
**Pain Symptoms**			
During active movements	65	0.3	49
During additional tests (passive opening, accessory movements, compression static pain)	6	0.4	59
During function (active movements and/ or additional tests)	89	0.7	69
During function and palpation	96	0.8	91
**Noises**			
During active movements	80	0.6	55
During additional tests	68	0.3	32
During function	77	0.5	60
**Restriction of movement**			
During active movements	92	0.6	10
During active movements and/or accessory movements	75	0.4	29

K = Cohen’s Kappa statistic in a TMD patient group (*n*= 79); TMD, temporomandibular disorder.
